# Correlation of FMR4 expression levels to ovarian reserve markers in *FMR1* premutation carriers

**DOI:** 10.1186/s13048-024-01425-0

**Published:** 2024-05-17

**Authors:** Ines Agusti, Maria Isabel Alvarez-Mora, Robin Wijngaard, Aina Borras, Tamara Barcos, Sara Peralta, Marta Guimera, Anna Goday, Dolors Manau, Laia Rodriguez-Revenga

**Affiliations:** 1 Clinical Institute of Gynecology, Obstetrics and Neonatology (ICGON), Hospital Clinic of Barcelona and FCRB-Institut de Investigacions Biomediques August Pi iSunyer (IDIBAPS), Barcelona, Spain; 2 Biochemistry and Molecular Genetics Department, Hospital Clinic of Barcelona and FCRB- Institut d’Investigacions Biomèdiques August Pi i Sunyer (IDIBAPS), C/Villarroel, 170, Barcelona, 08036 Spain; 3 CIBER of Rare Diseases, Instituto de Salud Carlos III, Madrid, Spain; 4 Department of Human Genetics, Donders Institute for Brain, Cognition and Behaviour, Radboud University Medical Center, Radboud University, Nijmegen, The Netherlands

**Keywords:** *FMR1* premutation, Fragile X-associated primary ovarian insufficiency, FMR4, Ovarian follicle reserve

## Abstract

**Background:**

Fragile X-associated primary ovarian insufficiency (FXPOI), characterized by amenorrhea before age 40 years, occurs in 20% of female *FMR1* premutation carriers. Presently, there are no molecular or biomarkers that can help predicting which *FMR1* premutation women will develop FXPOI. We previously demonstrated that high FMR4 levels can discriminate between *FMR1* premutation carriers with and without FXPOI. In the present study the relationship between the expression levels of FMR4 and the ovarian reserve markers was assessed in female *FMR1* premutation carriers under age of 35 years.

**Methods:**

We examined the association between FMR4 transcript levels and the measures of total antral follicle count (AFC) and serum anti-müllerian hormone (AMH) levels as markers of ovarian follicle reserve.

**Results:**

Results revealed a negative association between FMR4 levels and AMH (*r* = 0.45) and AFC (*r* = 0.64). Statistically significant higher FMR4 transcript levels were found among those *FMR1* premutation women with both, low AFCs and AMH levels.

**Conclusions:**

These findings reinforce previous studies supporting the association between high levels of FMR4 and the risk of developing FXPOI in *FMR1* premutation carriers.

**Supplementary Information:**

The online version contains supplementary material available at 10.1186/s13048-024-01425-0.

## Background

Primary ovarian insufficiency (POI) refers to the dysfunction or depletion of ovarian follicles with cessation of menses before age 40 years. Ovarian reserve markers (anti-müllerian hormone (AMH) and antral follicle count (AFC)) are related to the number of follicles in human ovaries; therefore, AMH and AFC have considerable value as diagnostic test for POI and highlight those at increased risk [1]. POI may be caused by many different factors including, chromosomal and genetic abnormalities, endocrinopathies, infectious processes or iatrogenic causes (anticancer treatments) [2]. While POI affects approximately 1% of women from the general population, it has been observed in 20% of women who carry the *FMR1* premutation [3]. POI associated with the *FMR1* gene premutation is referred to as Fragile X-associated primary ovarian insufficiency (FXPOI). The *FMR1* gene (OMIM*309550) contains a CGG repeat tract in the 5’ untranslated region. Based on the number of CGG repeats, four different *FMR1* alleles can be differentiated: normal, intermediate, premutation and full mutation [4–6]. While the full mutation (> 200 CGGs) is responsible for the fragile X syndrome (FXS), the leading single-gene cause of inherited intellectual disability, the *FMR1* premutation (55–200 CGG repeats) has been associated, among others pathologies, with FXPOI. Neither FXPOI nor diminished ovarian reserve is observed in women with full mutations [7], therefore, expanded CGG repeats within the *FMR1* premutation range have been proposed as the main pathogenic mechanism. *FMR1* premutation carriers have elevated levels of *FMR1* mRNA, and it is currently accepted that the excess expanded-repeat mRNA, per se, is pathogenic, leading to fragile X-associated pathologies (e.g. [8]).

FXPOI is an important health condition in which women do not only experience fertility problems, but are also at risk for other medical problems such as reduced bone mineral density, depression and anxiety problems, and increased risk of cardiovascular disease. Women with FXPOI should receive hormone replacement therapy until the typical age of natural menopause [9]. Currently, there are no biomarkers that help to predict those women that will develop a FXPOI. The risk for FXPOI has only been associated in a non-linear fashion with the CGG repeat size [10–12]; being those women with a *FMR1* premutation of approximately 80–99 CGG repeats the ones at the highest risk for FXPOI. In a previous study, we characterized the expression profiles of FMR4, FMR5 and FMR6 in female *FMR1* premutation carriers in order to determine a possible role in the pathogenesis of FXPOI and to investigate whether they could serve as a biomarker for the diagnosis of FXPOI [13]. FMR4, FMR5 and FMR6 are long-non coding RNAs (lncRNAs) originated from the *FMR1* gene locus that showed variable expression levels among *FMR1* premutation carriers, suggesting a functional association with fragile X-associated pathologies [14–17]. Our results revealed a significant association between FXPOI and high expression levels of FMR4, suggesting a potential role of FMR4 as a possible biomarker for FXPOI [13]. A limitation in the study design was that it was exploratory and, thus, *FMR1* premutation carriers with FXPOI enrolled had already developed ovarian dysfunction. In the present study, the relationship between *FMR4* expression levels and ovarian reserve markers was examined in young *FMR1* premutation female carriers under age of 35 years with regular menses (without FXPOI established).

## Materials and methods

### Study population

The present work is an extension of an earlier study on the prospective discriminatory capacity of FMR4 and other *FMR1* lncRNAs for FXPOI [13]. Based on the previous study of 36 *FMR1* premutation female carriers (20 with FXPOI and 16 without FXPOI), FMR4 expression levels > 12 increased the risk for developing FXPOI whereas FMR4 levels < 7 reduced the risk (*p* = 0.039). As previously reported in Alvarez-Mora et al. [13], while 30% of FXPOI women showed high levels of FMR4 expression, only 6% of women without FXPOI were found to have similar levels (supplementary Table 1).

I the present study, a total of 10 young female *FMR1* premutation carriers (CGG repeats between 55 and 200) were enrolled in order to validate the statistically significant distribution of FMR4 expression levels among *FMR1* premutation carriers with FXPOI and without FXPOI. All participants enrolled in the present study were recruited from fragile X syndrome families, reported normal ovarian function (regular cycles between 24 and 35 days) and none of them were under any hormonal contraceptive method. The women’s age range was 21–35 years old. None of them reported having smoking habits or alcohol use (< 1 day per week). The majority of the participants reported no use of oral contraception. The remaining discontinued the oral contraception by more than 5 years ago prior to this study. This study was approved by the Institutional Ethical Review Board of Hospital Clinic, Barcelona. All research was performed in accordance with relevant guidelines/regulations and in accordance with the Declaration of Helsinki (2013). All patients that were included in this study signed a written informed consent.

### Assessment of ovarian reserve

A peripheral blood sample was obtained and transvaginal ultrasonography and hormonal controls were routinely conducted. Serum anti-müllerian hormone (AMH) estimation was done by chemoluminescence immunoassay with paramagnetic particles for quantitative determination (AMH B13127 Beckman Coulter kit and in the ACCES2 device) (LOQ 0.02 ng/ml, interassay coefficient of variation < 5%; results expressed in ng/ ml). Normal level, corresponding to normal ovarian reserve, was considered over 1.1 ng/ml. Antral follicle count (AFC) was defined as the number of bilateral follicles (2–9 mm in diameter) in early follicular phase. AFC was done by scanning the ovary from the outer to the inner margin. A Voluson S6 unit, General Electrics Medical Systems (Austria), equipped with a 5–7 MHz vaginal probe were used. In this examination, a baseline gynecological assessment was performed to exclude gynecological pathology together with the AFC. Normal AFC was taken if it ≥ 7.

### DNA, RNA extraction and cDNA synthesis

A blood sample for determination of CGG repeat number, *FMR1* mRNA and FMR4 level was obtained from each subject. Genomic DNA and total RNA were isolated from 5 ml of peripheral blood by standard methods (Puregene and Purescript kits, Gentra). Total RNA isolation was performed from blood using the PAXgene® Blood RNA Kit (Qiagen) according to the manufacturer’s protocols. In order to determine the RNA concentration, a Qubit RNA IQ assay (ThermoFisher Scientific) was used. The RNA integrity was proved with the Bioanalyzer 2100 (Agilent). Using the High-Capacity cDNA reverse transcription Kit (ThermoFisher Scientific) and following the manufacturer’s instructions, cDNA was synthesized.

### *FMR1* CGG repeat size

CGG repeat analysis was determined using the AmplideX® PCR/CE FMR1 kit, following manufacturer’s recommendations (Asuragen).

### FMR4 and *FMR1* mRNA quantification

*FMR1* mRNA and FMR4 expression levels were quantified by digital droplet PCR (ddPCR) The *FMR1* mRNA assay was performed using the QX200™ ddPCR™. Pre-design TaqMan *FMR1* gene expression assay-FAM labeled was used together with the housekeeping gene GUSβ assay-VIC labeled (ThermoFisher Scientific, Santa Clara, California) to be run in a duplex reaction. FMR4 expression analysis was performed as previously described [13]. Primer sequences for FMR4 were extracted from Elizur and coworkers [17]. In order to normalize FMR4 copies relative to nuclear DNA, the *GAPDH* gene was used as reference gene. Results were analyzed with QuantasoftTM Software (Bio-Rad). The Poisson statistics was used to calculate target RNA concentrations. The expression of FMR4 was reported as (copies/cell) corrected for the expression of the reference gene *GAPDH*. Expression levels are shown as transcripts per ten thousand cells.

### Statistical analysis

The IBM® SPSS® Statistics software version 25 (SPSS, Chicago, USA) and the open-source computing environment R version 4.2 (R Foundation for Statistical Computing, Vienna, Austria) were used to carry out statistical analysis. Results were expressed as mean ± standard deviation (SD). Statistical significance of differences between means was examined using the parametric t-Test with Bonferroni correction. Significance was accepted for *P*-value < 0.05.

## Results

### Determination of the AMH, AFC and FMR4 expression levels in *FMR1* premutation carriers

The characteristics of the *FMR1* premutation carriers enrolled in the present study are presented in Table 1. The mean age was 31.9 ± 4.4 years and the mean body mass index (BMI) 21.92 ± 2.8 kg/m^2^. The mean CGG repeat size for the *FMR1* allele carrying the premutation was 74.5 ± 13.8 CGG repeats. Serum AMH levels and AFC were measured in all of them. In four cases AMH levels were < 1.1 ng/ml, indicating women with diminished ovarian reserve (case 1, 6, 8 and 9). The remaining 6 cases showed high AMH levels > 3 ng/ml, corresponding to normal ovarian reserves. Regarding AFC measurements among our samples, two participants showed very low AFC (< 7) (case 8 and 9), three presented with normal values 7–20) and 5 showed high values (> 20) (Table 1). FMR4 transcript and *FMR1* mRNA levels were also evaluated in total RNA extracted from peripheral blood by ddPCR (Table 1). The results obtained showed similar *FMR1* mRNA levels among all samples (1.03 ± 0.3), whereas the FMR4 transcript levels were variable among samples (8.24 ± 3.7), and within the previously reported ranges [13].

**Table 1:** Clinical and molecular characteristics of *FMR1* premutation carriers cases included in the study

ID	Age (years)	BMI (kg/m^2^)	AFC	AMH (ng/ml)	CGG Repeat size	FMR1 mRNA/cell	FMR4/cell
Case_1	29	22.3	9	0.37	29/59	0.98	5.2
Case_2	35	22.4	> 20	3.7	32/58	0.54	5.8
Case_3	35	20	20	5.44	20/55	1	8.3
Case_4	35	28	16	3.09	29/78	1.39	5.6
Case_5	21	18.2	> 20	4.49	30/70	0.83	7.5
Case_6	33	18.7	9	0.9	31/89	1.35	5.6
Case_7	35	21	> 20	4.9	33/81	1	5.3
Case_8	32	24.1	3	0.02	17/73	0.55	14.6
Case_9	34	23.1	1	0.24	30/91	1.58	15.0
Case_10	30	21.4	> 20	3.4	53/91	1.1	9.8

BMI: body mass index; AFC, antral follicle count; AMH, anti-müllerian hormone

### Association of FMR4 expression levels with AMH and AFC

**Fig. 1:**
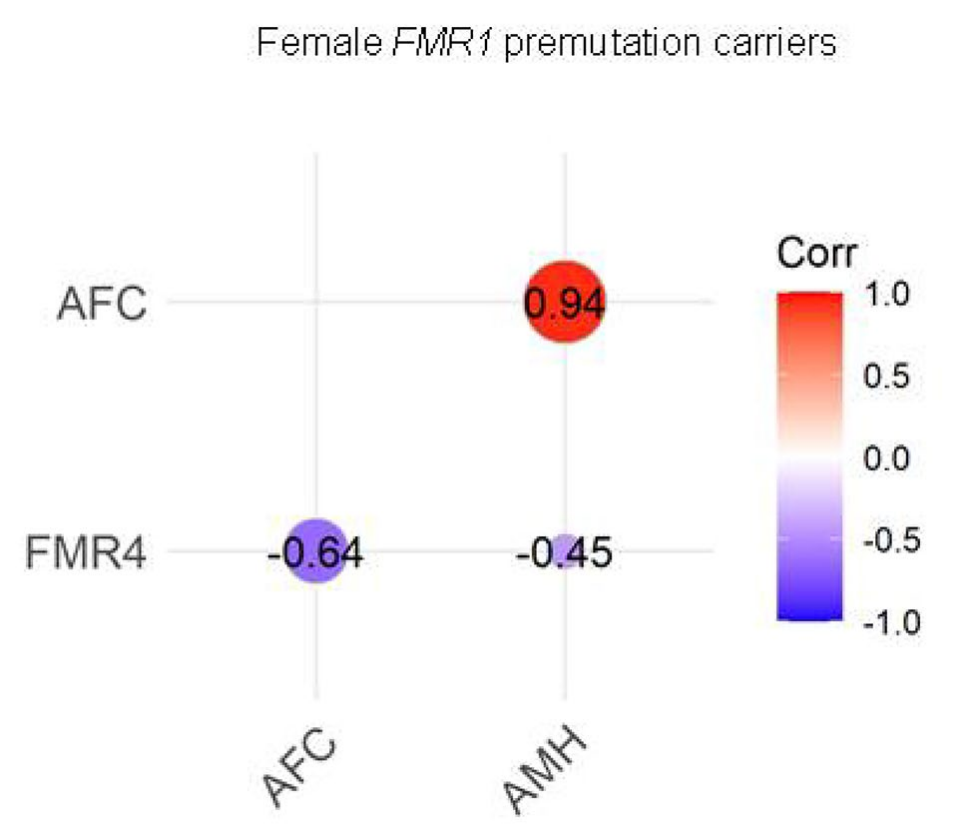
Correlation between serum AMH levels, AFC and FMR4 expression levels. Correlations were evaluated among total *FMR1* premutation carriers

Relations between AMH, AFC, and FMR4 transcript levels are shown in Fig. 1. As expected, high positive and significant correlation was found between AMH levels and AFC (*r* = 0.94; *P* < 0.001), meaning that patients with good ovarian reserve have high AMH and AFC values, while those with poor ovarian reserve have low levels. Contrary, a negative correlation was found between AMH, AFC and FMR4 (*r* = 0.45 for AMH and *r* = 0.64 for AFC). Although the correlation coefficients were moderate a barely significant correlation was found for FMR4 and AFC (*P* = 0.047), suggesting that FMR4 might help as an additional biomarker for FXPOI.

Case samples were further categorized into three groups according to the boundaries for the AMH levels and the AFC in the ovarian reserve test provided by the “Bologna criteria” to define low ovarian reserve [18]:

- Group A, AFC ≥ 7 and AMH ≥ 1.1 ng/ml, 6 women (both AFCs and AMH levels in the normal range);
- Group B, AFC ≥ 7 and AMH < 1.1 ng/ml or AFC < 7 and AMH ≥ 1.1 ng/ml, 2 women (normal AFCs and low AMH levels) or (low AFCs and normal AMH levels);
- Group C, AFC < 7 and AMH < 1.1 ng/ml, 2 women (low AFCs and low AMH levels).

**Fig. 2:**
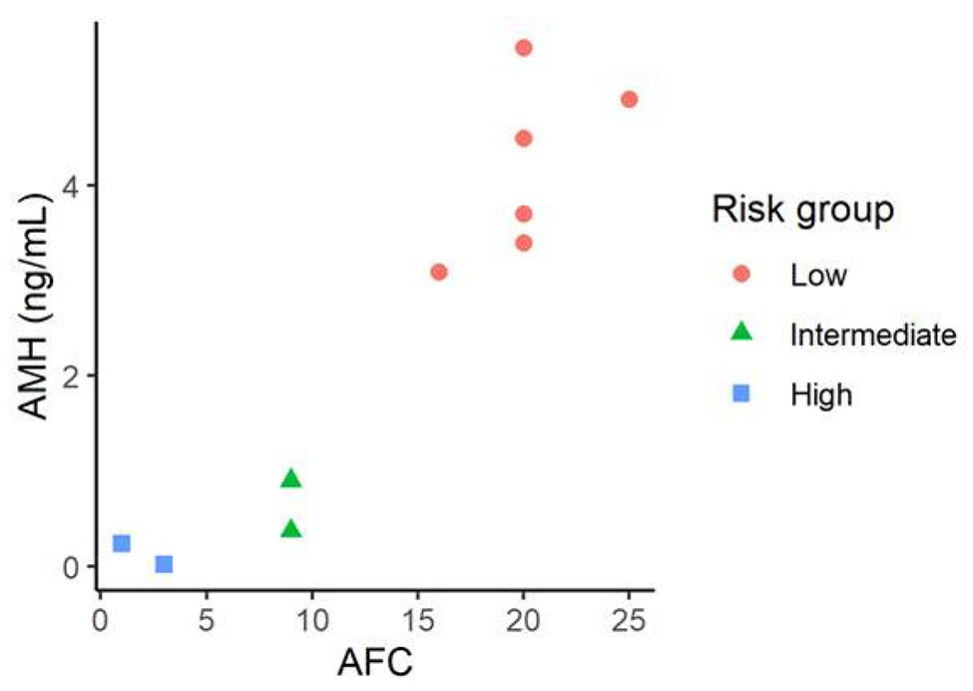
Case samples categorization. Case samples categorized into low, intermediate or high FXPOI risk group according to the boundaries for the AMH levels and the AFC in the ovarian reserve test provided by the Bologna criteria” (Ferraretti et al., [18])

While groups A and C showed concordant AMH levels and AFC, those within group B showed discordant values. In regard to the risk of developing FXPOI, group A could be considered as a low risk group, group B as an intermediate risk group, whereas group C could be considered as a high risk group (Fig. 2).

**Fig. 3:**
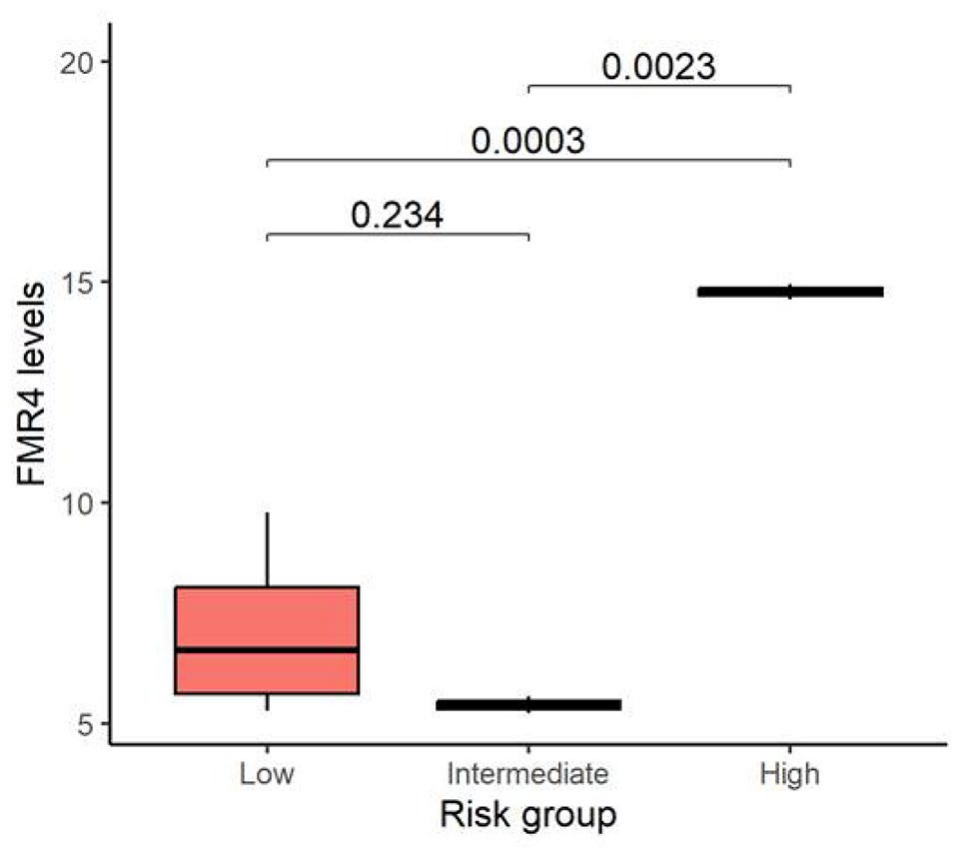
Expression levels of FMR4. FMR4 expression levels compared between low, intermediate and high FXPOI risk groups. Comparisons were statistically significant between high and both intermediate and low risk groups (*P* < 0.05)

In order to assess whether FMR4 levels could provide further information on the risk of developing FXPOI, transcript levels were measured and compared between groups (Fig. 3). Statistically significant differences were obtained when comparing FMR4 transcripts levels of those with high risk to those with both an intermediate (*P* = 0.0023) or low risk (*P* = 0.0003). Interestingly, based on the cut-off of FMR4 levels (> 12) for FXPOI described in our previous data [13], those women in group C who were at high risk based on AFC and AMH for FXPOI (cases 8 and 9) had FMR4 levels above this threshold.

### Diagnostic value of FMR4 for FXPOI

Since we previously showed a good diagnostic value of FMR4 for FXPOI, we compared predictability of FXPOI based on the AFC and AMH levels and the added value of considering FMR4 expression levels. As shown in Table 2, in those cases with an intermediate FXPOI risk based on AMH level and AFC, the value of FMR4 expression added discriminatory effect and helped to reclassify them as low FXPOI risk since FMR4 expression levels were under the cutoff of 7.

**Table 2:** Comparison of discrimination performance of risk models for predicting fragile X-associated primary ovarian insufficiency (FXPOI)

ID	FXPOI Risk Group based on AFC and AMH levels	FXPOI prediction risk model (Alvarez-Mora et al., 2022)	FXPOI Risk Group based on Model
Case_2	Low	0.42	Low
Case_3	Low	0.55	Low
Case_4	Low	0.38	Low
Case_5	Low	0.47	Low
Case_7	Low	0.36	Low
Case_10	Low	0.56	Low
Case_1	Intermediate	0.40	Low
Case_6	Intermediate	0.37	Low
Case_8	High	0.76	High
Case_9	High	0.76	High

## Discussion

The combination of AFC and AMH levels has been postulated as the markers with highest predictive value for early detection of POI [19]. While AMH is released in females by the ovarian granulose cells and is involved in initial follicle development [20], the AFC measures the number of antral follicles in the ovary, reflecting the number of follicles that will mature. AMH serum levels together with AFC correlate with ovarian follicle number, making them a reliable marker of ovarian reserve [21, 22]. Ovarian insufficiency is a continuum of impaired ovarian function. When the ovarian dysfunction starts, these ovarian reserve markers begin to deteriorate, ending up to low or undetectable levels once entered the POI stage. Recently, Jiao and coworkers [19] described that the combination of both markers was highly promising to predict early ovarian decline, as they showed a high sensitivity and specificity to detect different stages of ovarian insufficiency (normal ovarian reserve, pre-POI, early POI and POI).

Apart from Turner’s syndrome, the *FMR1* premutation is the most common known congenital cause of POI. Women with the *FMR1* premutation have a 20% risk of FXPOI, but nowadays, there are no molecular indicators that aid to predict it occurrence, leaving young *FMR1* premutation carriers without a personalized reproductive assessment. The causes of the incomplete penetrance of FXPOI are not well understood and, apart from the *FMR1* premutation, there are still some unknown genetic, epigenetic or environmental factors that might be influencing. In a previous study a significant association between FXPOI and high expression levels of FMR4 was revealed, suggesting a potential role of FMR4 as a possible biomarker for FXPOI. With a diagnostic power of 0.67, the ROC curve analysis showed that FMR4 can distinguish *FMR1* premutation carriers with FXPOI [13].

On the basis of these observations, in the current study, ovarian reserve indicators along with the FMR4 levels were characterized in young *FMR1* female premutation carriers in order to further evaluate their significance in predicting the risk of developing FXPOI. Pairwise correlations between AMH levels, AFC and FMR4 were assessed, and as expected, a strong significant correlation between AMH level and AFC was obtained (correlation coefficient = 0.94). Interestingly, the pairwise correlation between AMH levels, AFC and FMR4 levels showed a moderate negative association (-0.64 and − 0.45, respectively), suggesting the FMR4 might be further considered as an addition marker for predicting FXPOI (Fig. 1).

Although currently, no standardized AMH and AFC reference or cutoff value is available for pre-POI diagnosis we took the values described by Ferrareti et al. known as the “Bologna criteria” [18] and stratified the *FMR1* premutation carriers cohort into those with high, intermediate or low FXPOI risk based on serum AMH levels and AFC (Fig. 2). The majority of samples showed concordant AMH and AFC values, but 20% of them had discordant values. This percentage is in agreement with previously described data in clinical practice, where one in five women had discordance in the AFC and AMH level [23]. We further compared the mean FMR4 expression level among these 3 groups. Although caution must be taken due to the limited sample size, those with poor ovarian reserve markers, considered as the group with high FXPOI risk, showed statistically significant higher FMR4 levels and above the threshold determined in our previous study (Fig. 3). We further compared the risk of developing FXPOI obtained when considering AFC and AMH levels by the predicted probability of FXPOI based on our previously reported results that considered FMR4 expression levels [13]. Using a FMR4 expression levels cutoff of > 12 for high FXPOI risk and < 7 for low FXPOI risk, we can better discriminate those *FMR1* premutation carriers at risk of FXPOI (Table 2), especially in those cases where discordant AMH levels and AFC are detected. Nevertheless, a follow-up of this cohort is necessary in order to confirm our hypothesis.

In the general female population, *FMR1* premutation occurs with a relative high estimated frequency that range from 1 in 250 to 400 females [24]. The impact of being a female *FMR1* premutation carrier is enormous, not only because it challenges women’s fertility and lifelong health, but also because it increases the risk of having and offspring with fragile X syndrome. Although, currently, there is no way to prevent or reverse the impaired ovarian function associated with FXPOI, the fertility preservation field has rapidly evolved, providing alternative solutions to preserve fertility. Therefore emphasis should be placed on early identification of those *FMR1* premutation women at risk of developing FXPOI that can take advantage of these solutions, such as embryo and oocyte cryopreservation.

## Conclusions

In this study, we provide new evidences that, adding the FMR4 expression levels along with ovarian reserve markers, might help to better identify *FMR1* premutation carriers at risk of FXPOI. The main weakness of our study is the low number of cases enrolled as well as the lack of follow-up. Moreover, lifestyle factors such as smoking, alcohol use or BMI, are known to influence reproductive health. Whether these factors are also affecting FMR4 expression levels has not been explored. Nevertheless, the herein reported results warrant future prospective, longitudinal cohort studies to confirm the potential role of FMR4 as a FXPOI biomarker and to develop strategies for fertility improvement. We hope that these results will help to improve the management of *FMR1* premutation carriers, promoting a tailored approach to patient handling.

### Electronic Supplementary Material

Below is the link to the electronic supplementary material.

Supplementary Material 1

## Data Availability

The analyzed data sets generated during the study are available from the corresponding author on reasonable request.
