# Supplementary material for: Correlation of FMR4 expression levels to ovarian reserve markers in FMR1 premutation carriers
Source: J Ovarian Res. 2024 May 17;17:103. doi: 10.1186/s13048-024-01425-0 (PMC11100203; doi:10.1186/s13048-024-01425-0)
Supplement: Supplementary file 1 — Supplementary Material 1 [file 13048_2024_1425_MOESM1_ESM.docx]

Supplementary Table 1. Percentage of *FMR1* premutation female carriers with and without FXPOI based on FMR4 expression levels (modified from Alvarez-Mora et al., 2022).

|  | **CGG repeat (mean ± SD)** | **FMR4 expression level** | | | **p-value** |
| --- | --- | --- | --- | --- | --- |
|  |  | **1-7** | **7-12** | **>12** | **0.039*** |
| **FXPOI (n=20)** | 100 ± 35 | 10% | 60% | 30% |  |
| **No FXPOI (n=16)** | 88 ± 26 | 44% | 50% | 6% |  |

Significance: * p<0.05. The exact p-values were calculated with the Fisher exact test.
